# Perinatal Asphyxia and Its Associated Factors among Live Births in the Public Health Facilities of Bahir Dar City, Northwest Ethiopia, 2021

**DOI:** 10.1155/2021/3180431

**Published:** 2021-11-08

**Authors:** Magarsa Lami Dabalo, Simachew Animen Bante, Getahun Belay Gela, Selamawit Lake Fanta, Lemesa Abdisa Sori, Wondu Feyisa Balcha, Yomilan Geneti Muse, Tigist Derebe Tesfahun

**Affiliations:** ^1^School of Nursing and Midwifery, Haramaya University, College of Health and Medical Sciences, Harar, Ethiopia; ^2^Department of Midwifery, Bahir Dar University, College Medicine and Health Sciences, Bahir Dar, Ethiopia; ^3^Department of Nursing and Midwifery, Dire Dawa University, College of Health Sciences, Dire Dawa, Ethiopia

## Abstract

**Background:**

Birth asphyxia is a serious clinical problem of newborn babies, which occurs due to impaired blood-gas exchange and results in hypoxemia. Despite improvements in the diagnosis and management of perinatal asphyxia, it has become the leading cause of admission and neonatal mortality, especially in developing countries.

**Objective:**

This study was aimed at assessing factors associated with perinatal asphyxia among live births in the public health facilities of Bahir Dar city, Northwest Ethiopia, 2021.

**Method:**

Health facility-based cross-sectional study was employed from April 1-30/2021 in the public health facilities of Bahir Dar city among 517 mother-newborn pairs. The data were collected by systematic random sampling technique, entered by using Epi data 3.1, and analyzed using SPSS 25.0 version. Bivariate and multivariable logistic regression analyses were employed to estimate the crude and adjusted odds ratio with a confidence interval of 95% and a *P* value of less than 0.05 considered statistically significant. Frequency tables, figures, and descriptive summaries were used to describe the study variables.

**Result:**

In this study, 21.7% (95% CI: 18.2%–25.5%) of the newborns had perinatal asphyxia. Malpresentation (AOR = 4.06, 95%CI = 2.08-7.94), uterotonic drug administration (AOR = 2.78, 95%CI = 1.67-4.62), meconium-stained amniotic fluid (AOR = 4.55, 95%CI = 2.66, 7.80), night time delivery (AOR = 1.91, 95%CI = 1.17, 3.13), and preterm delivery (AOR = 3.96, 95%CI = 1.98, 7.89) were significantly associated with perinatal asphyxia. *Conclusion and Recommendation*. In the present study, the proportion of perinatal asphyxia was high. To mitigate this problem, there is a need to focus on early identification of the risk factors like fetal malpresentation, preterm labor/delivery, and managing them appropriately. Administering uterotonic drugs should be based on indication with close supervision.

## 1. Background

The perinatal period is the time extending from gestational age the fetus attains viability to the end of the seventh completed day of life [1]. Perinatal asphyxia happens when the baby receives too little oxygen because of the inability of the newborn to initiate and sustain adequate respiration immediately after delivery [2]. The Integrated Management of Newborn and Childhood Illness (IMNCI) in Ethiopia assigns a newborn to be asphyxiated if any of the following are seen: not breathing, gasping, poorly breathing (<30 breaths/min), and APGAR score < 7 at the first 5 minutes of delivery [2]. Despite improvements in diagnosis and management of birth asphyxia through an organized and well-equipped resuscitation program in recent years, perinatal asphyxia has become a foremost cause of admission and death in neonatal units especially in developing countries [3, 4].

Newborn suffering from serious perinatal asphyxia commonly has a poor muscle tone, cyanosis, poor responsiveness, perfusion, and respiratory effort, which result in a low five-minute APGAR score, and cardiac arrest and death can be caused by extreme degrees of asphyxia [5]. Perinatal asphyxia was the major complication, and it can lead to mental, social, and physical incompatibility because of severe hypoxic-ischemic organ damage in newborns [6]. Intrapartum hypoxic events may also result in stillbirth, neonatal, or postneonatal mortality, and neonatal encephalopathy is a strong predictor of long-term impairment [7].

Globally, perinatal asphyxia could be a common and serious newborn problem and it contributes to both neonatal morbidity and mortality [8]. According to WHO 2015 report, the 3^rd^ leading cause of under-five child deaths was perinatal asphyxia, which accounts for 11% next to complications of preterm birth (17%) and pneumonia (15%) of the top five leading causes of mortality including diarrhea and neonatal infections [3, 9, 10]. The global neonatal mortality rate ranges from as low as 0.9/1000 in developed nations to as high as 44.2/1000 in developing countries, including Ethiopia, in which the neonatal mortality rate constitutes 42% of under-5 deaths [11].

According to mortality estimates released by the World Bank Group, United Nations Population Division, and the WHO report of 2018, 5.4 million deaths occurs in the first five years of life. Newborns covered around 50% of the deaths, and most of them die due to preventable causes such as malaria, diarrhea, pneumonia, and complications during birth like perinatal asphyxia. In Africa, NMR is the highest when compared with another region, which accounts for 55/1000 live births, 5 times higher than that of European 10/1000 live births [12–14]. The research in Guinea showed that newborn deaths covered 30% of all deaths in Africa where the leading causes of death in newborns were perinatal asphyxia (41%) [15].

In Ethiopia, perinatal asphyxia is the first leading cause of neonatal mortality that constitutes 34% followed by preterm birth (25%) [16]. The majority of neonatal deaths in Ethiopia happen due to conditions related to neonatal asphyxia and prematurity, which contribute to around 60% of total neonatal deaths [3, 17]. Ethiopian Mini-Demographic Health Survey 2019 report on trends in early childhood mortality rates indicated that under-5 mortality rates decreased from 67/1000 live births in the 2016 EDHS to 55/1000 live births in 2019. Similarly, infant mortality declined from 48/1000 live births in 2016 to 43/1000 live births in 2019. However, NMR increased from 29/1000 live births in 2016 to 30/1000 live births in 2019 [18, 19]. Amhara Region was among the highest with NMR at the national level 47/1000 live births [19].

According to the reports of different studies conducted in Ethiopia, it was shown that the prevalence of perinatal asphyxia ranges from 12.5% to 32.8% [20, 21]. Different findings in different countries show that primiparity, complications during pregnancy like preeclampsia/eclampsia and antepartum hemorrhage (APH), intrapartum complication like fever, cord around fetal neck, umbilical cord prolapse, malpresentation, prolonged labor, prolonged rupture of membranes (PROM) and administration of uterotonic drugs, prematurity, low birth weight (LBW), maternal age, and medical complication were identified as risk factors of perinatal asphyxia [20–33], while a retrospective study done in Ghana shows that the use of partographs during labor was associated with a reduced incidence of birth asphyxia [31].

Despite Ethiopia's remarkable success in achieving the MDG-4 goals before five years, the decrement in NMR was comparatively low after that success and at the end of the MDG era, the government of Ethiopia is again running to address the SDGs to decrease NMR by 2030 to at least 12/1000 live births [12, 18, 19, 34, 35]. To achieve the SDG targets concerning maternal and child health, knowing and mitigating the risk factors of perinatal asphyxia are crucial to reduce neonatal mortality. Therefore, this study was intended to assess the proportion and associative factors of perinatal asphyxia with primary data in the public health facilities of Bahir Dar city, Northwest Ethiopia.

## 2. Methods

### 2.1. Study Design and Period

A health facility-based cross-sectional study design was employed from April 1 to 30/2021 at the public health facilities of Bahir Dar city.

### 2.2. Study Area

The study was conducted in the public health facilities of Bahir Dar city. Bahir Dar city is the capital city of the Amhara Region. The city is located approximately 565 km northwest of Addis Ababa, the capital city of Ethiopia. The city has a total population of 518,193 of which 265,156 are females. In the city, there are 3 governmental hospitals and 10 governmental health centers. They provide outpatient and in-patient department services including maternal and child health care [36–38].

### 2.3. Source Population

The source population was all newborns who had been delivered alive in the public health facilities of the Bahir Dar city administration.

### 2.4. Study Population

The study population composed of systematically selected newborns who had been delivered alive in selected public health facilities of the Bahir Dar City administration during the actual study period.

### 2.5. Inclusion and Exclusion Criteria

All newborns who had been delivered alive after viability in selected public health facilities of Bahir Dar City administration during the actual data collection period were included, while newborns with congenital anomalies (lethal congenital anomalies) incompatible with life after birth, referred immediately after delivery, or died within the first five minutes of delivery and newborn delivered from multiple pregnancies where one outcome was death of a twin were excluded.

### 2.6. Sample Size Determination

The sample size was calculated using a single population proportion formula by considering the following assumptions: proportion of perinatal asphyxia in Debre Tabor General Hospital was 28.35% [39] and *Zα*/2 is the critical value for normal distribution at 95% confidence level, which is equal to 1.96 (*Z* value of alpha at =0.05) or 5% level of significance (*α* = 0.05) and a 5% margin of error (*ω* = 0.05). The sample size was adjusted by adding a 10% nonresponse rate and design effects of 1.5; the final sample size was 517 mother-newborn pairs.

### 2.7. Sampling Procedure and Technique

A multistage sampling technique was done to select the study population. There are thirteen public health facilities in the city. All three hospitals were selected, and two health centers were chosen at random. The total sample size was proportionally allocated for each health facility of the administrative city based on six-month delivery report (June 28, 2020, to December 29, 2020). The numbers of delivery in six months were 1800, 2512, 1340, 780, and 690 in Tibebe Gion Specialized Hospital, Felege Hiwot Comprehensive Specialized Hospital, Addis Alem Primary Hospital, Bahir Dar Health Center, and Han Health Center, respectively. For each selected health facility of the city, the total sample size was proportionally allocated. The sample size after proportional allocation was 131, 182, 97, 57, and 50, respectively, for Tibebe Gion Specialized Hospital, Felege Hiwot Comprehensive Specialized Hospital, Addis Alem Primary Hospital, Bahir Dar Health Center, and Han Health Center. Then, eligible mother-newborn pairs in each facility were selected by using a systematic random sampling technique based on their average monthly delivery. The average number of monthly delivery in all selected (five) health facilities was 1187. The sampling fraction or *K*^th^ units were calculated by dividing total monthly delivery in selected health facilities by the sample size of the study (1187/517 = 2). The starting unit was selected by using the lottery method among the first *K*^th^ units in each facility.

### 2.8. Dependent Variable

The dependent variable was perinatal asphyxia.

### 2.9. Independent Variables

Maternal sociodemographic factors are as follows: mother's age, occupation, marital status, educational level, family monthly income, and religion.

Maternal antepartum obstetrics factors are as follows: pregnancy status, gravidity, ANC visit, number of ANC visits, obstetrical ultrasound, bad obstetric history, and complications during pregnancy.

Maternal medical factors are as follows: diabetes mellitus, anemia during pregnancy, malaria, smelly or excessive vaginal discharge, and chronic hypertension.

Intrapartum factors are as follows: fetal presentation, mode of delivery, method of vaginal delivery, uterotonic administration, duration of labor, PROM, duration of ROM, duration of 2^nd^ stage of labor, complication during labor, condition of Amniotic fluid, partograph, time of delivery, labor attendant, cord around the neck, and birthplace.

Neonatal factors are as follows: newborn sex, gestational age, birth weight, and size for GA (birth size).

### 2.10. Operational Definitions

Perinatal asphyxia is defined as a newborn considered to have birth asphyxia when its 5^th^-minute Apgar score was under 7 [2].

Prolonged labor is when failure to the progress of labor was considered when the labor exceeds 12 hours in primigravida or 8 hours in multipara mothers after the latent first stage of labor was diagnosed [40].

Prolonged second stage is diagnosed when nulliparous mothers had pushed for 3 hours or above and for multipara mothers 2 hours or above, if epidural anesthesia is used, and if nulliparous women pushed for 4 hours or above and for multipara mothers for 3 hours or above [40].

Prolonged rupture of the membrane is a rupture of membrane or leaking for >12 hours, if membrane ruptures before onset of labor, if the rupture of the membrane is after the onset of labor, and it is leaking for >18 hours [41].

Income category is when based on average monthly income in ETB, it is categorized as ≤2000, 2001–5000, and >5000 [42].

### 2.11. Data Collection Instrument

Data was collected from study participants by using a pretested structured questionnaire. The instrument was adapted from relevant works of literature developed for a similar purpose by different authors [6, 30, 43] and modified to the local context. The questionnaire was developed in English and translated into Amharic by an individual who has good ability of these languages, then retranslated back into English to check for its consistency. The questionnaire consists of five main parts: maternal sociodemographic factors, antepartum obstetric-related factors, maternal medical factors, intrapartum factors, and neonatal factors.

### 2.12. Data Collection Tools and Procedures

The data were collected by ten diploma and BSc midwives and supervised by two BSc midwives. The data collector's responsibility was to fill the questionnaire after getting the written consent of the study subjects by interviewing the mother of the newborn and diagnosing birth asphyxia based on the Apgar score. The mother was interviewed after her condition and vital sign were stable after delivery, the maternal medical records were then inspected to collect information on the antenatal period, and also, the newborn was observed by the data collectors.

### 2.13. Data Quality Assurance

Data was collected by trained data collectors, and pretesting of the instrument was done before the actual data collection. The questionnaire was pretested before the actual data collection period on 5% [26] mother-newborn pairs getting delivered at study cities, to check the conceptuality of the data. Data collectors and the supervisors were trained for two days by the investigator on how to observe deliveries, APGAR scoring, a clinical sign of perinatal asphyxia, and how to fill the data. After necessary modifications and correction were done to standardize, its reliability (coefficient of Cronbach's alpha was 0.81) and consensual validity had been ensured and additional adjustments were made based on the results of the pretest. The completeness of the data was checked by data collectors during data collection, and daily supervision was done for data completeness by supervisors.

### 2.14. Data Processing and Analysis

The data were entered into Epi data 3.1; edited and cleaned for inconsistencies, missing value, and outliers; and then exported to SPSS version 25.0 for analysis. During analysis, all explanatory variables which had an association in bivariate analysis with a *P* value < 0.25 were entered into a multivariable logistic regression model, and enter method was used to get the AOR and those variables with 95% of CI, and a *P* value of < 0.05 was considered statistically significant with perinatal asphyxia. By using the variance inflation factor, a multicollinearity test was done and no collinearity exists between the independent variables. The model goodness of the test was checked by Hosmer-Lemeshow goodness of the fit, and its *P* value was 0.78. Frequency tables, figures, and descriptive summaries were used to describe the study variables.

## 3. Results

### 3.1. Sociodemographic Characteristics

A total of 517 mothers who gave live birth were interviewed with a 100% response rate. The median age of the mothers was 26.00 years with an interquartile range [22, 29] and ranging from 18 to 48 years. Among the study participants, 313 (60.6%) were housewives. Regarding educational status, 168 (32.5%) mothers had no formal education. The mean participants' monthly income was 3623.04 ETB with ±SD = 3102.73, and 438 (84.7%) of respondents were Orthodox Christianity Religion followers.

### 3.2. Antepartum and Medical Factors

In this study, 303 (58.6%) mothers were multigravida and 509 (98.5%) had singleton pregnancy status. Of the mothers, 102 (19.7%) had a bad obstetric history at least one time and 478 (92.5%) mothers had attended at least one ANC visit. About 83.0% (*n* = 430) of mothers had an ultrasound check-up at least one time, and 64 (12.4%) had faced obstetric complications during the current pregnancy. In our study, 26 (5%) of mothers had a history of medical illness, and anemia had accounted for 18 (69.2%) of the illness.

### 3.3. Intrapartum Factors

In the present study, 458 (88.6%) fetal presentation was vertex and 405 (78.3%) of the mothers had a vaginal delivery. Of the mothers, 338 (65.4%) in labor were not administered uterotonic drug and 465 (89.9%) gave birth within the normal duration of labor and delivery. Of the mothers, 52 (10.1%) had a history of PROM and among mothers who entered the second stage of labor, 47 (10.6%) had their second stage of labor prolonged. Of the mothers, 332 (68.7%) were followed with partograph and 498 (96.3%) had no complication during labor and delivery. About 52% (*n* = 269) of the mothers gave birth at night and 267 (51.6%) of the labor were attended by midwives alone. One-tenth (*n* = 52) of the babies had the cord around the neck during delivery, and 410 (79.3%) of the mothers delivered at the hospital.

### 3.4. Neonatal Factors

Of the newborns delivered, 264 (51.1%) were females and 468 (90.5%) were delivered at GA ≥ 37 weeks. Of the newborns, 452 (87.4%) birth weight was ≥2500 grams, and the mean birth weight was 2896.88 grams. From delivered neonates, 460 (89.0%) had their weight at birth appropriate for their gestational age.

### 3.5. Proportion of Perinatal Asphyxia

The proportion of perinatal asphyxia was found to be 112 (21.7%) (95% CI: 18.2%–25.5%). From asphyxiated neonates, 91 (81.3%) and 21 (18.8%) had moderate and severe perinatal asphyxia, respectively.

### 3.6. Factors Associated with Perinatal Asphyxia

In bivariate analysis, gravidity, obstetric and medical complication, fetal presentation, mode of delivery, uterotonic drug administration, meconium-stained amniotic fluid, delivery time, labor-attendant, cord around fetal neck, gestational age, and birth weight at delivery had *P* value less than 0.25 (Table 1).

Neonates born with malpresentation were 4.1 times more likely risky to develop birth asphyxia compared with newborns delivered with vertex presentation (AOR = 4.06, 95%CI = 2.08-7.94), and those mothers who were administered uterotonic drug, their newborns are 2.8 times more likely to be asphyxiated relative to those newborns who were delivered without uterotonic drug administration (AOR = 2.78, 95%CI = 1.67-4.62).

Neonates born from mothers with meconium-stained amniotic fluid were 4.6 times more likely to develop perinatal asphyxia than neonates delivered from mothers with clear amniotic fluid (AO = 4.55, 95%CI = 2.66-7.80). Newborns who were delivered at night time were 1.91 times more likely prone to develop birth asphyxia than those delivered in the daytime (AOR = 1.91, 95%CI = 1.17-3.13), and neonates delivered at gestational age < 37 weeks were 4 times more likely to have birth asphyxia than neonates delivered at ≥37 weeks of gestational age (AOR = 3.96, 95%CI = 1.98-7.89) (Table 2).

## 4. Discussion

In this study, the proportion of perinatal asphyxia among live births in Bahir Dar city's public health facilities was 21.7% (95% CI: 18.2–25.5%). The finding is in line with a study conducted in Gosau, Nigeria (21.1%) [43]. The proportion of perinatal asphyxia in this study was higher than in the studies conducted in Nigist Eleni Mohammed Memorial Hospital (15.1%), in Ayder comprehensive specialized hospital in Tigray (18%), and in Jimma Medical Center (18%) [26, 27, 44]. This difference might be due to differences in methodology and/or differences in the study setting and differences in maternal service given from place to place.

Furthermore, the proportion of perinatal asphyxia in this study was lower than that in studies done at Debre Tabor General Hospital (28.3%), Wolaita Sodo (25.7%), and Dilla University Referral Hospital (32.8%) [21, 39, 45].

This might be due to fact that this study was done at both the health center and hospital level, but those studies were done at the hospital level; an example study done at Debre Tabor was done at the level of a general hospital only; at the hospital level, more complicated cases were anticipated including perinatal asphyxia. However, the finding in this study is higher than that in a study conducted in Jimma zone public hospitals (12.5%) [20]. The possible reason for this difference might be the study setting, as this study was conducted by including both the health center and hospitals, while the study in the Jimma zone was conducted only in the hospital setting.

In the present study, intrapartum and fetal factors were significantly associated with perinatal asphyxia. Neonates born with malpresentation were 4.1 times more likely to develop perinatal asphyxia. This finding is in line with studies done in different parts of Ethiopia (in Debre Tabor General Hospital, in Asalla Teaching and Referral Hospital, and in Jimma zone public hospitals) [20, 39, 46] and with also a study conducted in Cameroon [24]. The reason behind this was the fact that fetal malpresentation like breech presentation increases the risk of trauma during delivery, fetopelvic disproportion, which is risky for prolonged labor, and the occurrence of cord prolapse also increased in fetal malpresentation; those all increase the incidence of perinatal asphyxia [47].

For those mothers who gave birth by the administration of uterotonic drug, their newborns were 2.8 times more likely to be asphyxiated. This finding was agreed with studies done in India and Bangladesh [23, 32, 48]. This might be because uterotonics can cause hyperstimulation (tachysystole) and decrease blood flow to the fetus through the placenta [47]. The protocol of oxytocin infusion is also different from facility to facility. Furthermore, most of the time uterotonics were administered after the labor was prolonged for a different reason and both factors increase the happening of birth asphyxia [49].

Neonates born from mothers with meconium-stained amniotic fluid were 4.6 times more likely to suffer from perinatal asphyxia. This study finding was congruent with other studies done in different parts of Ethiopia [20, 21, 26–28, 39] and also with a study done in Kenya [25]. The explanation might be because meconium-stained amniotic fluid may cause mechanical obstruction to airways after being inhaled during intrapartum period. Dysfunction of surfactant caused by meconium, as well as its direct toxicity, also causes tissue inflammation, necrosis, and hypoxia, which in turn stimulates colonic activity by increasing intestinal peristalsis and loosening the anal sphincter and results in passage of meconium, which increases the risk of developing birth asphyxia [47, 50].

Newborns who were delivered at night time were 1.91 times more prone to develop perinatal asphyxia. This finding agrees with the study done at Debre Tabor General Hospital [39]. This might be since relatively fewer health care providers are hired to follow laboring mothers and also to attend the delivery with a high burden at night time. The higher likelihood of perinatal asphyxia during the night time may also be due to relatively longer decisions to the delivery interval at night than in daytime during emergency C/S [51]. Furthermore, it may also be due to consultation hierarchy and sometimes consulted senior delayed arrival for difficult labor at night time, which may have played its role.

Neonates delivered at preterm were 4 times more likely to have perinatal asphyxia than neonates delivered at ≥37weeks. This study finding was consistent with other studies in Ethiopia [26, 28, 46, 52] and also with a study in Northern Tanzania [53]. The reason could be due to complications of preterm birth of the newborn like respiratory distress syndrome that results from immature lungs that are not competent to sustain necessary oxygenation resulting to hypoxia that causes neurological damage like cerebral palsy and necrotizing enterocolitis [47].

### 4.1. Limitation

In this study, perinatal asphyxia was diagnosed only based on the 5^th^-minute Apgar score because of the lack of facilities for arterial blood gas and umbilical cord blood pH analyses in the study setting.

### 4.2. Conclusion and Recommendation

In the present study, the proportion of perinatal asphyxia was high. Fetal malpresentation, uterotonic drug administrations, meconium-stained amniotic fluid, delivery at night time, and preterm delivery were significantly associated with perinatal asphyxia. Therefore, it is important to encourage women to have ANC visits, because this warns to identify the risk factors of prenatal asphyxia like fetal malpresentation and preterm labor/delivery, and this in turn helps to manage them appropriately. In the intrapartum period, the health care providers also have to follow the labor attentively, especially at night time by assigning one health care provider for one laboring mother. Additionally, administering uterotonic drugs should be based on indication with close supervision by focusing on appropriate implementation of induction/augmentation protocol at the facility level. Further study is also needed on perinatal asphyxia by considering arterial blood gas analysis and pH of umbilical cord blood to mitigate this fatal problem of newborn babies.

## Figures and Tables

**Table 1:** Bivariate logistic regression analysis for perinatal asphyxia among neonates delivered alive in the public health facilities of Bahir Dar city, Northwest, Ethiopia, 2021 (*n* = 517).

Variables	Birth asphyxia		COR (95% CI)
	Yes	No	
Gravidity			
Primigravida	58	156	1.71 (1.13, 2.61)
Multigravida	54	249	1
Obstetric complication			
Yes	24	40	2.49 (1.43, 4.34)
No	88	365	1
Medical complication			
Yes	10	16	2.38 (1.05, 5.41)
No	102	389	1
Fetal presentation			
Mal presentation	29	30	4.37 (2.49, 7.67)
Vertex	83	375	1
Mode of delivery			
C/S	33	79	1.72 (1.07, 2.77)
Vaginal	79	326	1
Uterotonics			
Yes	62	117	3.05 (1.99, 4.69)
No	50	288	1
Amniotic fluid condition			
Meconium stained	496	49	5.65 (3.50, 9.12)
Clear	63	356	1
Delivery time			
Night	73		1.99 (1.29, 3.08)
Day	39	196	1
Labor attendant		209	
Midwife with IESO	26	51	1.97 (1.02, 3.81)
Midwife only	51	216	0.91 (0.53, 1.59)
Midwife with resident	12	49	0.95 (0.43, 2.07)
Midwife & obstetrician	23	89	1
Cord around the fetal neck			
Yes	17	35	1.89 (1.02, 3.52)
No	95	370	1
Gestational age			
<37 weeks	26	23	5.02 (2.73, 9.24)
≥37 weeks	86	382	1
Weight at birth			
Low birth weight	25	40	2.62 (1.51, 4.55)
Normal birth weight	87	365	1

**Table 2:** Multivariable logistic regression analysis for perinatal asphyxia among neonates delivered alive in the public health facilities of Bahir Dar city, Northwest, Ethiopia, 2021 (*n* = 517).

Variables	Birth asphyxia		AOR (95% CI)	*P* value
	Yes	No		
Gravidity				
Primigravida	58	156	1.54 (0.94, 2.52)	0.084
Multigravida	54	249	1	
Obstetric complication				
Yes	24	40	0.93 (0.43, 2.45)	0.929
No	88	365	1	
Medical complication				
Yes	10	16	1.16 (0.41, 3.30)	0.780
No	102	389	1	
Fetal presentation				
Mal presentation	29	30	4.06 (2.08, 7.94)	0.001^∗^
Vertex	83	375	1	
Mode of delivery				
C/S	33	79	1.34 (0.67, 2.66)	0.411
Vaginal	79	326	1	
Uterotonics				
Yes	62	117	2.78 (1.67, 4.62)	0.001^∗^
No	50	288	1	
Amniotic fluid condition				
Meconium stained	49	49	4.55 (2.66, 7.80)	0.001^∗^
Clear	63	356	1	
Delivery time				
Night	73	196	1.91 (1.17, 3.13)	0.010^∗^
Day	39	209	1	
Labor attendant				
Midwife with IESO	26	51	1.51 (0.68, 3.32)	0.310
Midwife only	51	216	1.20 (0.63, 2.28)	0.583
Midwife with resident	12	49	0.62 (0.25, 1.54)	0.302
Midwife & obstetrician	23	89	1	
Cord around the fetal neck				
Yes	17	35	1.68 (0.78, 3.64)	0.185
No	95	370	1	
Gestational age				
<37 weeks	26	23	3.96 (1.98, 7.89)	0.001^∗^
≥37 weeks	86	382	1	
Weight at birth				
Low birth weight	25	40	1.11 (0.44, 2.79)	0.824
Normal birth weight	87	365	1	

∗ indicates significance at a *P* value of <0.05.

## Data Availability

All related data have been presented within the manuscript. The data set supporting the conclusion of this article is available from the corresponding author (Magarsa Lami) upon reasonable request.

## Abbreviations

ANC: Antenatal clinic care
AOR: Adjusted odds ratio
APH: Antepartum hemorrhage
C/S: Cesarean section
CI: Confidence interval
COR: Crude odds ratio
EDHS: Ethiopian Demographic and Health Survey
IMNCI: Integrated management of newborn and childhood illness
LBW: Low birth weight
MDG: Millennium development goals
NMR: Neonatal mortality rate
PROM: Prolonged rupture of membranes
SDG: Sustainable development goal
SPSS: Statistical Package for Social Science
UNICEF: United Nations Children's Fund
WHO: World Health Organization.
